# From context to contexting: professional identity un/doing in a medical leadership development programme

**DOI:** 10.1111/1467-9566.13007

**Published:** 2019-10-23

**Authors:** Mathilde A. Berghout, Lieke Oldenhof, Wilma K. van der Scheer, Carina G. J. M. Hilders

**Affiliations:** ^1^ Erasmus School of Health Policy and Management Erasmus University Rotterdam Rotterdam The Netherlands; ^2^ Erasmus Centre for Healthcare Management Erasmus University Rotterdam Rotterdam The Netherlands

**Keywords:** Netherlands, medical leadership, professional identity, identity work, medical leadership development programme, physicians

## Abstract

Physicians are known for safeguarding their professional identities against organisational influences. However, this study shows how a medical leadership programme enables the reconstruction of professional identities that work with rather than against organisational and institutional contexts to improve quality and efficiency of care. Based on an ethnographic study, the results illustrate how physicians initially construct conflicting leadership narratives – heroic (*pioneer*), clinical (*patient's guardian*) and collaborative (*linking pin*) leader – in reaction to changing organisational and clinical demands. Each narrative contains a particular relational‐agentic view of physicians regarding the contexts of hospitals: respectively as individually shapeable; disconnected or collectively adjustable. Interactions between teachers, participants, group discussions and in‐hospital experiences led to the gradual deconstruction of the heroic –and clinical leader narrative. Collaborative leadership emerged as the desirable new professional *identity*. We contribute to the professional identity literature by illustrating how physicians make a gradual transition from viewing organisational and institutional contexts as pre‐given to *contexting*, that is, continuously adjusting the context with others. When engaged in contexting, physicians increasingly consider managers and directors as necessary partners and colleague‐physicians who do not wish to change as the new ‘anti‐identity’.

## Introduction

Physicians are well‐known for safeguarding their professional and *elite* identity upon ‘external threats’, such as the increase in managerialism and market logics in health care (Numerato *et al*. 2012). Managerialism, resulting in increased standardization, control, auditing and administration burdens, is said to threaten physicians' professional identity as it would hamper them from performing the essence of their work: treating patients (ibid.). Contemporary health care in Western countries, however, does not only face ‘external’ pressures but also ‘internal pressures’, such as the expansion of chronic diseases and multi‐morbid patients, cost‐efficiency objectives and shifts in care delivery from hospital to primary care. (Noordegraaf 2011). Increasingly, physicians are expected to adapt their work practices accordingly and pro‐actively shape organisational changes in their field. Yet critical insights in *how* professionals interpret these institutional pressures and how they reconstruct their own professional identities accordingly are still lacking.

In particular, a number of opinion‐making physicians seem to embrace these requests, as is reflected by their recent pleas for *medical leadership* (Berghout *et al*. 2017, Swanwick and McKimm 2011, Warren and Carnall 2011). By framing physicians as leaders, opinion‐makers stimulate other physicians to disrupt ‘old’ professional values, such as professional autonomy, hierarchy and socialization, in order to construct a *new medical identity*, which enables physicians to meet societal and clinical challenges (Berghout *et al*. 2018). In this light, various initiatives to develop medical leadership have emerged. New competency models complement technical skills with ‘leadership’ skills (ibid.). In addition, educational institutes have been established by medical associations offering physicians the possibility to train themselves as ‘leaders’ in medical leadership development programmes (MLDP) (Frich *et al*. 2015). These initiatives stimulate physicians to act as ‘leaders’ in their daily work by setting up multidisciplinary collaboration, taking care of cost‐efficiency and fulfilling roles in hospital governance.

Although ideologically framed as ‘the solution’ for contemporary issues in health care (Swanwick and McKimm 2011, Warren and Carnall 2011), the advocacy for medical leadership reflects complex and paradoxical identity demands. First, advocates stimulate physicians to get ‘back in the lead’ by regaining professional dominance in health care while simultaneously having to denounce professional values such as autonomy and hierarchy in order to become multidisciplinary *team players* (Berghout *et al*. 2018). Second, denouncing professional values such as professional autonomy, hierarchy and strong socialization processes is arguably easier said than done among highly socialized and institutionalized professionals (Freidson 2001, Reay *et al*. 2017). And third, medical leadership discourses presume the possibility of easily incorporating organisational demands into daily clinical practices. Although a number of scholars have shown that organisational and professional logics have become increasingly intertwined (McGivern *et al*. 2015, Noordegraaf 2011, Sheaff *et al*. 2003), many physicians still perceive these requirements as *competing* demands (Berghout *et al*. 2018).

In this study, we investigate the identity work – ‘the active construction of an individuals' identity’ (Pratt *et al*. 2006: 237) – that is carried out by physicians who identify themselves as ‘medical leaders’. Empirically, we zoom in on a medical leadership development programme (MLDP). We understand physician's participation in a MLDP as a need to reconstruct their medical identities to meet contemporary societal and clinical challenges. Investigating these identity processes in a MLDP is relevant as it provides critical insights in how professionals subjectively interpret conflicting institutional pressures and how they reconstruct their own professional identities accordingly (Bévort and Suddaby 2016, Reay *et al*. 2017). In the literature, MLDPs are recently considered as important identity spaces (Petriglieri and Petriglieri 2010) that enable identity work. By using the concept of *contexting* – continuously adjusting the organisational context with others (Asdal and Moser 2012, Bévort and Suddaby 2016, McGivern *et al*. 2015), we show how physicians aim to un*do* (Nicholson and Carroll 2013) their often‐assumed stable and detached professional identity by reinterpreting a new relational‐agentic view regarding organizational and institutional contexts and other (non) clinical actors.

### Identity, identity work and physicians

Theoretically, we draw on social constructivist accounts of identity, specifically focusing on the construction of professional identities (e.g. Alvesson and Willmott 2002, Brown 2015, Carroll and Levy 2010, Sveningsson and Alvesson 2003, Sveningsson and Larsson 2006). Identity refers to the social construction of the self and seeks to provide answers to questions such as ‘who am I (are we)?’ and ‘where do I (we) stand for?’ (Sveningsson and Alvesson 2003: 1164). Conceptually, a distinction can be drawn between social identities (nationality or gender) or personal identities (intelligence or height) (Brown 2015: 23) and *identity* in general that refers to “the meaning that an individual attach reflexively to *‘the self’*” (Brown 2015: 23). Following Alvesson and Willmott (2002), we use the concept of identity as the latter form, and we understand identity thus as fluid, dynamic and socially constructed rather than stable and static. Importantly, scholars point to the fact that identities are shaped by the self *and* the other. As Berger and Berger (1972: 62) put it: ‘only if an identity is confirmed by others it is possible for that identity to be real to the individual holding it’ (cited in Van Bochove and Burgers 2019). Identities are thus temporal constructions, which are ‘regularly constituted, negotiated and reproduced in social interactions’ (Sveningsson and Larsson 2006: 206).

Organisational change or tensions could decrease one's sense of a coherent identity and trigger *identity work* which refers to the engagement of individuals in ‘forming, repairing, maintaining, strengthening or revising the constructions that are productive of a sense of coherence and distinctiveness’ (Sveningsson and Alvesson 2003: 1165). Yet this does not necessarily mean that actors by definition seek a coherent sense of self. Rather, individuals can identify with multiple, contradictory selves (Ahuja *et al*. 2017, Beech *et al*. 2008). Switching between different selves can, for example, be a strategic act as it allows one to balance between different logics or objectives (Iedema *et al*. 2004).

In this study, we specifically build on the professional identity literature. Professional identity refers to ‘an individual's self‐definition as a member of a profession and is associated with the enactment of a professional role’ (Chreim *et al*. 2007: 1515). Professional identification is different than other types of social identification as it is established through selection, prolonged training, socialization and self‐regulation and underpinned by professional autonomy and values (Freidson 2001). Professional identity is thus determined by what one does instead of where one works (e.g. organisational membership) (Chreim *et al*. 2007, Pratt *et al*. 2006, Spyridonidis *et al*. 2015). In contrast to ‘other’ identities, professional identity is often considered as relatively stable and detached from organizational contexts. Physicians are well‐known for safeguarding their professional identity after ‘identity violations’ that potentially threaten their social status and professional autonomy, such as the increase in managerial logics and the presence of non‐clinical actors in health care (Currie *et al*. 2012, Doolin 2001). The underlying assumption in these studies is that physicians do not want to fundamentally change their professional identity and remain oppositional towards organizational and institutional contexts and non‐clinical actors.

However, increasing evidence criticizes the assumption that professional identities are stable and in opposition to the organizational context and instead shows that professional identities are becoming more fluid, hybrid and blurred with organizational contexts (Ahuja *et al*. 2017, Bévort and Suddaby 2016, Kyratsis *et al*. 2017, McGivern *et al*. 2015, Noordegraaf 2011, Reay *et al*. 2017, Spyridonidis *et al*. 2015). The concept of ‘hybridity’ emphasizes the mediation between professional and organizational logics and illustrates that ‘organizing’ is in fact an intrinsic part of professional work. Likewise, physicians are potentially active agents who engage with organizational contexts in order to deal with contemporary healthcare challenges instead of detaching themselves to safeguard their ‘old’ professional identity. Studies following this line of reasoning, illustrate how physicians, increasingly confronted with cost‐efficiency objectives, reforms, chronically ill patients and multi‐morbidity, in fact unravel and adjust parts of their professional identity in order to adapt to their ‘new realities’ (Kyratsis *et al*. 2017, McGivern *et al*. 2015, Reay *et al*. 2017). For example, Reay *et al*. (2017) show how physicians increasingly interpret themselves as ‘head of teams’ instead of ‘autonomous experts’ (p. 1064). Likewise, McGivern *et al*. (2015) argue how ‘hybrids’ – physicians in managerial roles – challenge and disrupt institutionalized professionalism to align their professional identities to ‘new’ managerial contexts.

In a similar vein, we aim to illustrate how physicians, by means of participating in a MLDP, try to un*do* (Nicholson and Carroll 2013) their often‐assumed stable professional identity by reinterpreting their relational position towards hospital contexts to better deal with perceived institutional pressures. We show that physicians not only adapt to new realities and organizational contexts by reinterpreting their professional identities but that they increasingly interpret themselves as active agents who co‐adjust these contexts with others, which we label as ‘contexting’ (Asdal and Moser 2012, Bévort and Suddaby 2016, McGivern *et al*. 2015). Given the increasing hybridity of professional work and identities, it is especially interesting to investigate *how* physicians give meaning to ‘contexts’ and construct their relational‐agentic position towards ‘the context’ (e.g. organizational (hospital) contexts, institutional contexts and other (non‐clinical) actors.

### Leadership programmes as identity workspaces

Numerous scholars have linked leadership to identity processes (Andersson 2015, Carroll and Levy 2010, Ford 2006, Gagnon and Collinson 2014, Martin and Learmonth 2012, Nicholson and Carroll 2013). These studies interpret leadership as a discursive phenomenon which is socially constructed and aim to demonstrate its performative effects on identities (Sveningsson and Larsson 2006). Various studies show how actors strategically use leadership rhetoric to reconstruct professionals' identities by prescribing preferred ‘leadership’ competencies and practices, thereby steering professionals' behaviour and attitudes (Berghout *et al*. 2018, Carroll and Levy 2010, Ford 2006, Martin and Learmonth 2012). Similarly, Sveningsson and Larsson (2006) argue that leadership can be considered as a ‘symbolic attribute’ which can be mobilized in identity work (ibid.: 208). They show how contemporary leadership discourses offer individuals a more appealing identity, one that is associated with charismatic and transformative visionaries, in contrast to management, which is laden with negative values such as bureaucracy and slow reforms.

In addition to studies of leadership development in organisations, a small number of scholars have begun to study LDP's as ‘identity workspaces’ (Carroll and Levy 2010, Gagnon and Collinson 2014, Nicholson and Carroll 2013, Petriglieri and Petriglieri 2010) which can be broadly understood as spaces that enable and stimulate individuals' identity work. Most research on Leadership Development Programmes (LDP) is underpinned by positivistic notions of leadership and mainly focus on competency development, ignoring the social, political and organisational contexts where LDPs are situated in (Carroll and Levy 2010). In contrast, social constructivist approaches to LDP's aim to study collective processes in professional identity construction, which is a relatively understudied phenomenon in research on professional identity reconstruction (Reay *et al*. 2017). In line with other authors (Pouthier *et al*. 2013, Reay *et al*. 2017), we argue that studying these collective processes is highly relevant to understanding identity work because identity un/doing is rarely an individual process; rather identity comes into being through ongoing interaction and collective discussions concerning who we are and where we stand for.

By using the term ‘identity undoing’ (Nicholson and Carroll 2013), we aim to show that LDP's are not only merely spaces where actors work on *new* identities but also spaces where identities are ‘destabilized, unravelled and deconstructed’, influenced by power relations, individual and collective actions and discursive forces. We will show how a MLDP enables the deconstruction – ‘undoing’ – of professional identities and reconstruction – and ‘doing’ – of ‘new’ professional identities through collective identity work. In line with Sveningsson and Larsson (2006) we thus understand ‘leadership’ and the leadership narratives articulated by the MLDP participants as symbolic attributes in performing their identity work.

## Methods

We conducted an ethnographic study of a 1‐year MLDP (September 2017–July 2018). Ethnographic methods allowed us to get a deep understanding of people's attitudes, beliefs and self‐perceptions with the aim to understand the subjects of study ‘from within’, in people's own terms and frames (Willis and Trondman 2000).

The MLDP is developed and led by a Dutch university‐affiliated centre for education in healthcare governance and management. The general aim of the programme is ‘to enable physicians to take the lead in the continuous improvement of healthcare’. The programme consists of six collective sessions (total of 9 days) and three 2‐hour in‐house hospital sessions. In addition to the collective sessions, every participant carried out a hospital‐based improvement project (see Appendix 1 for project examples and content of the programme). The progress of the projects was discussed during the in‐house sessions.

Participants are 23 physicians (six hospitals) representing 13 different medical disciplines. The programme was guided by 4 facilitators and 15 guest speakers. Three sub‐sessions and all in‐house sessions were attended by six hospital directors. Participants were chosen by the hospital board (n = 19) or applied for a position in the programme (n = 4). Although the majority was encouraged to participate, participation was in the end voluntarily and driven by physicians' affinity with leadership. Most participants were early‐career professionals wanting to do more than ‘the clinical’. A few participants held formal managerial positions, for example, a part‐time position as head of a clinical department or director of the daily medical staff board. All participants and directors were first informed by e‐mail about the possibility of participating in this study and subsequently briefed by the first author at the start of the programme. Afterwards, all participants were asked for their consent, which was given by all participants. During the first two sessions, participants asked the first author several questions related to data collection and analysis. Thereafter, the presence of the first author was perceived as ‘normal’ and did not raise further questions. This was confirmed by the facilitators of the programme.

The data consist of > 200 pages of field notes, retrieved from around 100 hours of observations. The observations were guided by several ‘sensitizing concepts’ (Bowen 2006): identity, medical leadership and hybridity. Participants' relation to context (e.g. hospital context, colleagues, board of directors, managers) emerged as an important theme early in the programme and steered the first author in the subsequent observations. The programme did not entail explicit sessions on ‘identity’ but contained many sessions/discussions related to participants' current and future roles in health care – for example about who they are as physicians and where they want to stand for – which we labelled as ‘identity work’.

In addition to the observations, informal interviews were held with participants, trainers and guest speakers during coffee breaks, breakfast, lunch, dinner or evening drinks to get a deeper understanding of participants' and facilitators' perceptions on the issues discussed. The data collection was conducted by the first author, who joined all collective sessions and six (50%) of the in‐house sessions in five hospitals. The third author joined half of the collectives sessions and all in‐house sessions. During the in‐house sessions she was responsible for the introductory meeting. The development of the in‐house sessions was discussed afterwards with the co‐authors. During the programme, the third author facilitated an introductory presentation in the first session about leadership styles. Participants were asked to collectively discuss what medical leadership entails. This stimulated participants to reflect upon their professional roles and identities. In the last module, the first author presented the preliminary findings as a member check to verify if the interpretations of these findings related to those of the participants. This was confirmed by the participants. Additionally, documents were analysed, including programme brochures, presentation slides and learning materials.

We analysed our data by investigating the different social constructions of medical leadership by participants in interaction with other participants, facilitators, hospital directors and guest speakers. We started our analyses with a first phase of inductive, or ‘open’ exploration of the data, which revealed three leadership narratives (heroic, clinical, collaborative; see Appendix 2). Identities often take the form of narratives, which can be understood as the stories we construct of ourselves in addressing questions such as who am/are I/we? or where do I/we stand for? (Beech *et al*. 2008: 52; Brown 2015: 23) and identity work then thus reflects ‘the processes through which people develop narratives of the self’ (Beech *et al*. 2008: 52). Important to note is that we understand narratives as the *means* to articulate a (preferred) identity. Yet, for an identity to be real for the one holding it, others need to confirm and validate the narrative and thus the identity (Berger and Berger 1972). Physicians were not exclusively bound to one narrative but could shift between the narratives constructing diverse, or even contradictory, selves. Signifiers of the narratives are discussed in Appendix 3.

In the first phase of inductive coding a dominant tension between working with or against ‘the context’ emerged as an important theme. By ‘context’ we mean actors (e.g. peers, non‐clinical colleagues, hospital directors, managers), organizations (e.g. hospitals, healthcare insurance companies) or institutions (e.g. the healthcare inspectorate, the Dutch healthcare system). In making sense of their role as medical leaders, participants continuously referred to ‘the context’ that either stimulated or hindered the development or execution of their leadership roles. Important to note is thus that we use to term ‘context’ to illustrate how participants refer to actors, organizations or institutions and as such ‘context’ can thus have multiple meanings. In the second phase we linked the narratives to the tension between working with or against ‘the context’. Each narrative revealed a distinctive position towards organisational and institutional contexts: heroic (individually shaping the context), clinical (disconnected from the context), collaborative (collaboratively adjusting the context).

In the third and final phase we analysed the process of how identities are collectively made and unmade over the course of the programme. By using the concept of ‘identity un/doing’ (Nicholson and Carroll 2013) we revealed the de/construction of participants' professional identities through collective identity work.

## Results

The results show how physicians performed identity work in a medical leadership development programme by constructing different leadership narratives of the self: that is, the heroic, clinical or collaborative leader. Each leadership narrative contains a particular relational‐agentic view of physicians regarding *the context* of hospitals: respectively as shapeable by an individual heroic leader; as disconnected from the clinical leader or as collectively adjustable by collaborative leadership. The results reveal how over the course of the programme, interactions between teachers and participants, group discussions and in‐hospital experiences contributed to the gradual and partial deconstruction of the heroic and clinical leader narratives. In particular, their relational‐agentic view regarding ‘the context’ was deconstructed: that is, individualistic notions of agency [heroic narrative] and detached standpoints vis‐a‐vis the hospital context and organisational actors [clinical narrative]. In addition, the results demonstrate the rebuilding of a new identity of a ‘collaborative leader’ who collectively adjusts and reshapes organisational and institutional contexts by working across disciplinary and organisational boundaries. We reflect on the tensions between different leadership narratives and the consequences of these narratives for the reconfiguration of professional work in contemporary hospitals.

### Entering the leadership programme: different leadership narratives and relations to context

During the first two sessions of the leadership programme, which entailed introductory rounds and discussions about the meaning of medical leadership, participants collectively discussed their aspirations to become medical leaders in their hospitals. We observed how participants expressed themselves in different and contradictory ways as *heroic, clinical and/or collaborative leaders* (see Appendix 2). Initially, participants understood themselves primarily as heroic or clinical leaders and just occasionally as collaborative leader. A collaborative understanding of leadership was gradually constructed over the course of the programme.

#### The heroic narrative: individually shaping the context

The heroic narrative shows the construction of a heroic leader, *‘the pioneer’*, for whom being a physician is more than ‘just’ the clinical. The heroic leader has a strong vision about future health care and feels it is her/his responsibility as a physician to play a role in this by individually reshaping the existing organisational context: both financially and in terms of quality. Participants felt the urge to step up as ‘leaders’ who can ‘radically transform health care’ as they argued that current hospital and healthcare contexts are ‘not‐innovative’, ‘conservative’ and ‘in lack of financial resources’, thereby obstructing quality and efficiency improvements. When mobilizing the heroic leader narrative, participants seemed to interpret themselves as the driving force in change processes, individually *shaping a new context*, for example, by reorganizing clinical work or developing innovative medical apps.

Participants producing these narratives presented themselves in contrast to those who ‘just come and go’, ‘lack an innovation mind’ or are ‘unwilling to change’, thereby granting themselves much agency in comparison to others. Others (physician‐colleagues) were often blamed in explaining why innovations – ranging from simple daily processes in care delivery to larger, multidisciplinary improvement projects – failed as they were framed as ‘unwilling to change’, ‘pursuing different interests’ and ‘bad communicators’. Through this discursive opposition, participants aimed to make the leader identity exclusive for a ‘happy few’, which would enable themselves to step forward as visionary and heroic leaders: *‘It is as if everyone still thinks the world is flat, while I already know for a long time the world is round’* (respondent 8). Many interpreted the leadership programme as a means to deal with colleagues ‘who do not want to change’ and to learn ‘how to cope with frustration’ and to ‘keep going’. Interestingly, this narrative showed that physician‐colleagues, and not managers, were perceived as the new ‘anti‐identity’.

#### The clinical narrative: disconnected from the context

The clinical narrative displays the construction of *‘a ‘patients’ guardian’*, who aims to improve patient care and bring health care back to its essence: caring for patients. Participants expressed their needs to be this ‘self’ as they felt that health care was dominated and regulated by ‘outsiders’, for example, managers, the government, healthcare insurance companies, resulting into an exhausting amount of administration and an excessive focus on costs rather than quality of care: *‘The hospital is not ours [physicians] anymore. There are managers, a business director. And things aren't going very well’* (respondent 20). The clinical narrative suggested that physicians perceived the patient's and their own position as under threat and therefore felt they needed to step up as ‘leaders’ by safeguarding health care from interference of outsiders.

This narrative came into being by a continuous process of de‐identification from ‘different‐others’ like managers and hospital directors with a background in business administration. Participants argued that improving patient care is something that only professionals (physicians/nurses) can do because of their unique link with the patient that managers lack: *‘we really feel what people go through in the consultation room’* (respondent 14). By emphasizing a boundary between clinical and non‐clinical professionals, participants aimed to preserve the clinical leader identity for an exclusive group. Within the clinical narrative this distinctiveness was further underscored by arguing that as a physician ‘you don't work for the organisation’ since ‘that is not your primary concern’ (respondent 24), thereby further *disconnecting themselves from managerial and organisational contexts* which were interpreted as pre‐given barriers.

#### The collaborative narrative: collectively shaping the context with others

The collaborative narrative shows the construction of a leader as *‘linking pin’*, who aims to bring actors together and balance different interests in order to ensure quality *and* cost‐efficiency of care. Care delivery is considered as a co‐production between different actors who have equal responsibilities for the quality of care. Collaborative leaders transcend disciplinary or organisational boundaries, thereby considering the collaborative identity inclusive for more than just a ‘happy few’. Within this narrative, being a physician means being a responsible and accountable multidisciplinary team‐player who places the patient central instead of her/himself. Participants argued that this sense of self is required to deal with the increase in multi‐morbid patients, chronic diseases and shifts in care delivery from hospital to primary care.

The construction of a collaborative leadership narrative entailed a particular view as to how actors saw themselves in relation to ‘the context’ and ‘others’. Rather than viewing the hospital context as disconnected from themselves [clinical leader] or individually shapeable [heroic leader], the collaborative narrative shows how the context was interpreted as collectively adjustable and an as an important resource for change. Participants shaped their sense of self in relation to similar‐others (physicians) and different‐others (e.g. managers, business controllers or directors) by repeatedly reminding each other of the interdependencies in the delivery of care: ‘you have to do it together’ (respondent 2, 6, 24).

#### Conflicting leadership narratives

Initially, participants shifted between narratives adapting parts of the heroic/clinical/collaborative narrative, or expressed themselves merely through one narrative. Moreover, the following interaction illustrates how participants constructed different and at times *conflicting* narratives. Participants discussed what medical leadership means to them during the first day of the programme:

You are an excellent doctor and therefore you will lead others. (Heroic narrative; respondent 9)

No one says I want to be a medical leader to increase productivity, you don't act from an economic perspective. (Clinical narrative; respondent 16)

First comes the work floor, then finance and then profits. That is what makes us special. (Clinical narrative; respondent 9)

For me medical leadership means collaboration, knowing the perspectives from different actors. (Collaborative narrative; respondent 10)

What distinguishes a medical leader is the patient. You don't primarily work for the organization, that's not your primary concern. (Clinical narrative; respondent 24)

But the organization must function properly. (Collaborative narrative; respondent 2)

It's a service you deliver, optimal patientcare is also the organisation's concern (collaborative narrative; respondent 20)

A medical leader needs to transcend disciplines. You come from a certain blood type but you need to leave behind your own specialty. You need to develop a vision that is broader than your own little club. (Collaborative narrative; respondent 8)

[…]

The hospital is not ours [physicians] anymore. There are managers, a business director. And things aren't going very well (Clinical narrative; respondent 20).

[14 September 2017 module 1]

The excerpt shows how participants adapted different parts of the narratives (heroic and clinical narrative, respondent 9, and collaborative and clinical narrative, respondent 20). Moreover, the quotes illustrate how narratives could conflict. By employing the clinical narrative, participants (16, 9 [second quote], 24) emphasized the importance of disconnecting from the context to increase quality of care. Several participants (10, 2, 8) reacted by expressing a collaborative understanding of leadership and underscored the importance of adapting to the new organizational context and presence of others.

### Identity undoing: tensions and deconstruction

Over the course of the programme, participants experienced ‘identity violations’ (Pratt *et al*. 2006) in the enactment of their preferred leader identities: a mismatch between participants' self‐images and the perception of participants by others. Specifically *heroic* and *clinical* self‐perceptions led to tensions in daily practices and in improvement projects. In response to these experienced tensions, hospital directors, facilitators, guest speakers and co‐participants gradually deconstructed individualistic notions of agency [heroic narrative] and detached standpoints vis‐a‐vis the hospital context and organisational actors [clinical narrative] to stimulate different understandings of leadership.

First, participants experienced that it was extremely hard to engage others in adopting their view on necessary change [heroic narrative] and to get others on board due to competing interests or a lack of time and support. This hindered them to perform their role as medical leaders:

You receive a letter of the board of directors that you are a medical leader, but the rest of the hospitals thinks, what are you!? (Respondent 6)

You can only be a leader when others grant you that role. It is difficult for us, we don't get any extra education, money or time. […] You can't be a leader on an uninhabited island (respondent 9).

Participants expressed their frustrations about their lack of mandate and formal authority, comparing themselves to a ‘general without an army’ (respondent 7). Several physicians experienced uncertainty about how others perceived them as a leader, which led to questioning their sense of self:

I wonder … I am part of the daily medical board, do I see myself as a leader? I don't know … but I do encounter people reaching to me … do they do so because I have a link with ‘above’ or because they like me? […] Do you have followers because you have a mandate, or because they trust you? (Respondent 20; 15 September 2017, module 1)

Second, participants discussed that ‘outsiders’ – managers, politicians, healthcare insurance companies – increasingly determined physicians' work, for example, the duration of consults, type of care delivered or the use of quality standards. Participants were especially dissatisfied with an excessive presence of managers in their hospitals. They argued that managers often hamper change because of their budget constraints and focus on costs rather than quality. They therefore tried to bypass or avoid them. However, avoidance was a difficult strategy to maintain. Requests from ‘outsiders’ for insights into quality and efficiency of care in the development of change projects made it impossible for participants to keep their disconnected selves intact [clinical narrative].

In response to these experienced tensions, hospital directors, facilitators, guest speakers and co‐participants gradually deconstructed heroic self‐images and anti‐manager identities to stimulate different understandings of leadership. They criticized the physician's reluctant and oppositional attitude towards others – peers, managers, politicians, healthcare insurers or directors – and their relational‐agentic views regarding ‘the context’ as being individually shapeable [heroic narrative] or disconnected [clinical narrative] (see Appendix 3)

To illustrate this collective deconstruction process we provide an excerpt from an interaction between participants and a facilitator. During the 4th module, participants were split up in subgroups to discuss how to engage physicians in quality improvements. The following excerpt shows a collective discussion afterwards and illustrates the experienced tensions, subsequent critique and deconstruction of heroic and clinical understandings of leadership:

The problem is, if you want to change something, the answer you get is ‘write a business case’. (Respondent 2)

Pff, I recognize this. No one who says: ‘well that's a good idea.’ (Respondent 15)

Yes ok, but how do we turn this around? What is our advice? (Respondent 14)

I think the problem is that we as physicians are too little involved in quality issues. So it's imposed by the board and then often resistance arises. We shouldn't blame the board of directors but we should blame ourselves. The medical staff should be more motivated to increase quality of care. (Respondent 20)

Shouldn't we move towards a participation model? […] So that you have mandate in the boardroom and get medical staff involved in decisions. We need a medical staff board with formal authority. (Respondent 7)

But didn't we allow this to happen? We act as if this suddenly happens to us. (Respondent 14)

Quality standards are imposed upon us because we didn't make them ourselves. If we stand up and say ‘this is what we define as quality of care’ then no one has to tell us what to do. (Respondent 24)

“But what is your common purpose? What you are saying, has been said by physicians over the last 20 years. You don't need to become a manager. Do you really need formal authority to have impact?” (Facilitator 1)

(1 February 2018; module 4)

As the quotes show, respondents experienced tensions in their heroic and clinical understandings of leadership: ‘outsiders’ hamper proposed improvement objectives [clinical narrative] and a lack of formal authority obstructs engagement of medical peers [heroic narrative]. Fellow participants and a facilitator responded by deconstructing their relational‐agentic views towards the context, for example, ‘others’: instead of blaming others for obstructing quality improvement, physicians should increase their own participation in the formulation of quality standards.

### Identity doing: the construction of the collaborative leader

The experienced tensions and subsequent critique during group discussions and in‐house sessions led by many participants to a reconstruction of their sense of self as a *collaborative leader*. This was not an isolated but a relational process steered by co‐participants, facilitators, guest speakers and hospital directors.

First, participants were stimulated to reinterpret change as a *continuous* and *collaborative* process instead of an individual or isolated process. Hospital environments were repeatedly reframed as shared contexts that require collaboration from different stakeholders to solve mutual problems:*“[We] all have to face the same challenge: how can we improve healthcare through efficient use of resources? Collaboration and mutual trusts are key. Therefore, this program brings together all stakeholders: together with hospital directors, medical staff directors and participating medical specialists we discuss issues that are relevant in both the consultation – and board room”* (programme's brochure).

Instead of blaming others, participants were encouraged to find ‘common ground’, reconsider their ‘circle of influence’ and to learn how to build consensus. Guest speakers were carefully chosen depending on their [political] position (facilitator 1) (i.e. the inspectorate, health care insurers, the director of a collaboration between ‘top clinical hospitals’ and the director of The Dutch Council for Public Health and Society). In this way, facilitators aimed to teach participants that these actors have to become their allies instead of their opponents (facilitator 1). The following quote exemplifies a shift from being reluctant towards others to valuing common ground:

I have grown as a person. I agreed with the merger [between two locations of a hospital, to which she was reluctant at first]. It is difficult to act from your own optimal vision … A part of this is letting go, finding common ground. I found it very difficult to let go of my own opinion. I'm afraid that something is less good, but it is needed to let the process succeed. (Respondent 5, in‐house session, 17 January 2018)

Second, participants increasingly understood themselves as team players who collaborate across disciplinary and professional borders instead of individual professionals. To stimulate reflections on their professional identities and relation towards others and the organizational context, the programme included several self‐reflective sessions. During a boxing training (participants received sports boxing training and boxing against a partner was meant as a metaphor for ‘difficult’ conversations), participants gained insights in how they related to others:

I developed a broader perspective, broader than my own specialty, in which collaboration is really essential. […] The boxing session was really a moment of reflection on your own way of communicating and on perspectives of others. […] Collaboration, everyone knows it is important, but you are now more focused on its value. […] I think reflection and listening to each other are of real importance. Using change theories not to push your own ideas. (Respondent 2, in‐house session, 19 June 2018)

In addition, the following interaction during a session on innovation illustrates how a participant expressed a collaborative understanding of leadership that values being a team player over pursuing individual aims:

“Team interest is always more important than individual interest. […] If someone is an excellent chef, but a total asshole, then he has to leave. […]” (Guest speaker 4)

I'm in a conflict model with my project right now. [laughs] So what I'm getting out of this session is that from this conflict we can go into two directions. I have to work on establishing a relation with others, and then work towards innovation together. No more muddling through and then proceeding in the same old way. (Respondent 5, module 3, 8 December 2017)

Third, participants expressed an understanding of leadership that moves beyond excelling in clinical work. Optimizing care delivery processes and cost‐efficiency were increasingly valued as an important part of being a ‘modern’ physician:

We as physicians are used to focus merely on the clinical, searching for explanations. But now I realize how important the entire process around care delivery is and to involve others in your ambitions to realize change. (Respondent 10, in‐house session, 18 June 2018)

Incorporating cost considerations into daily practices was at first perceived as controversial by several participants [clinical narrative]. However, through group discussions and deconstructions (see Appendix 3) most participants increasingly valued the importance of acquiring knowledge into cost issues. Participants realized that for the success of their improvement projects it was necessary to demonstrate cost‐efficiency by developing a business case. The following two quotes illustrate the development of a participant's perception towards her responsibility in optimizing cost‐efficiency of care:

I agree that things could be more efficient … But if you don't know how much something [care] costs then you can't make it cheaper. No one has any idea in my department. […] And sorry if I keep complaining, but we as physicians we don't have any insights in these things right … neither does the hospital. (Respondent 11, module 5)

I became more critical, for example after that sessions about hospital finances I really got started, I wanted to get more insights into how this works … (Respondent 11, in‐house session 19 June 2018)

Moreover, participations increasingly valued collaboration with other non‐clinicians as they began to realize that they could share their responsibilities in optimizing care processes and cost‐efficiency with others (e.g. business managers and supporting staff).

#### Tensions in being a collaborative leader in practice

Although the construction of the collaborative self was a key development in the MLDP trajectory, it was not always a smooth transition when physicians returned to their own hospital. Physicians particularly experienced a lack of support from peers and hospital administrators with regard to their project and personal developments. In addition, they were not always granted the extensive time required for executing improvement projects because the daily pull of clinical work was perceived as too strong. This arguably hindered some physicians from wholeheartedly embracing their preferred identity as *collaborative leader* as the following quote illustrates:

I experienced difficulties in finding my role. You're not a medical manager, you're not part of the (hospital/medical) board. So what's your role then? But there's expected a lot from you. You receive no formal support or feedback while you do need that. (Respondent 10, z in‐house session 18 June 2018)

A lack of support by others led to identity violations as this obstructed some participants to be their preferred collaborative self. These identity violations caused participants stress and work dissatisfaction and hindered some participants from fully realizing their collaborative ambitions.

## Discussion

This paper examined the identity processes among hospital‐physicians participating in a Medical Leadership Development Programme. We showed how participants performed identity work as a means to deal with institutional pressures for change – for example, demands of affordability, efficiency, quality, patient‐centred care and task distribution – in contemporary hospital settings by constructing three different leadership narratives: that is, heroic (*pioneer*), clinical (*patient's guardian*) and collaborative (*linking pin*). Each leadership narrative contains a particular relational‐agentic view of physicians regarding *the context* of hospitals: respectively as shapeable by an individual heroic leader; as disconnected from the clinical leader or as collectively adjustable by collaborative leadership.

Early on in the MLDP, most of the performed narratives of leadership portrayed heroic or clinical identities. However, in the enactment of their heroic or clinical self, participants experienced identity violations: ‘others’ did not engage in adopting their view on necessary change [heroic narrative] and ‘outsiders’ increasingly influenced professional work [clinical narrative]. In response to these tensions, facilitators, guest speakers, directors and fellow participants gradually deconstructed individualistic notions of agency [heroic narrative] and detached standpoints vis‐a‐vis the hospital context and organisational actors [clinical narrative].Through collective discussions the ‘collaborative leader’ emerged as a desirable alternative: that is, someone who collectively adjusts organizational and institutional contexts by working across disciplinary and organisational boundaries.

Our results contribute to professional identity literature (Kyratsis *et al*. 2017, McGivern *et al*. 2015, Pouthier *et al*. 2013, Reay *et al*. 2017) that shows how physicians respond to institutional pressures and ‘identity violations’ (Berghout *et al*. 2018, Chreim *et al*. 2007, Spyridonidis *et al*. 2015). We illustrate how physicians, in interaction with others, unravelled their often‐assumed stable and detached professional identity through a reinterpretation of their position towards the ‘context’: other clinical and non‐clinical actors, organizations and institutions. By using the concept of ‘contexting’ (Asdal and Moser 2012), we demonstrate how physicians reinterpreted hospital contexts as collectively adjustable rather than as pre‐given settings that hinder them in pursuing their aspirations to increase quality and efficiency of care. Individualistic notions of agency [heroic narrative] and detached standpoints vis‐a‐vis organisational and institutional contexts (e.g. hospital settings, the healthcare system, healthcare insurance companies) and organisational actors [clinical narrative] were gradually deconstructed. In turn, by constructing a collaborative sense of self, participants aimed to break down institutionalized contexts in health care (e.g. boundaries between primary care and hospital care; disciplines; care/cure) to enable multidisciplinary teamwork and improvement of quality and efficiency of care.

During the course of the MLDP, participants increasingly identified with organisational objectives and non‐clinical actors, showing the hybridity of their professional identities and confirming recent findings of other scholars (Kyratsis *et al*. 2017, McGivern *et al*. 2015, Numerato *et al*. 2012, Reay *et al*. 2017). Specifically, participants perceived the optimization of care processes and cost‐efficiency as an important part of being a physician. However, the desirable identity as collaborative leader was not always easy to enact when physicians returned to their own hospital (e.g. when implementing their improvement project) as others (e.g. peers, directors) did not always support this new self‐image. Our findings reveal that when ‘new’ (organisational) responsibilities, such as multidisciplinary collaboration, are not backed‐up by a supportive environment this may lead to identity violations causing stress and work dissatisfaction. Although the MLDP offered an important supportive space to discuss these identity violations, physicians also needed a supportive space within the hospital environment itself to not become ‘isolated’ leaders with unrealized collaborative ambitions.

Moreover, our study reveals the influence and *importance* of ‘others’ in professional identity work. Participants were neither passive recipients of disciplining techniques imposed by facilitators, known in literature as identity regulation (Alvesson and Willmott 2002), nor individual directors of their own identity scripts. Rather, we observed how identity work was conducted in a *collective* manner. ‘Others’, educational instructors, directors, fellow participants, were actively engaged in identity work by stimulating participants to investigate their professional values and relation to the changing hospital context. Professional identity reconstruction is thus not merely an individual conduct regulated by professionals but others including *non*‐clinicians can play a significant role in changing the professional self (Chreim *et al*. 2007).

Our findings confirm the importance of relating to or differentiating from others (different/similar) in identity work (Andersson 2015: 85, Brown 2015). The discourse of medical leadership was used by the participants to de‐identify from other physicians (heroic narrative) and managers (clinical narrative) or to actually identify *with* these similar/different others (collaborative narrative). Physicians are well known for their ongoing struggles with ‘others’ over power (Suddaby and Viale 2011). Accordingly, our findings illustrate how managers were still partly considered as the ‘anti‐identity’ in the heroic and clinical narrative (Martin and Learmonth 2012, Numerato *et al*. 2012). However, more interestingly, the collaborative leadership narrative shows that managers, other professions and directors, were increasingly regarded as equal and necessary partners in health care while colleague‐physicians who refuse to collaborate in change initiatives were considered as the new ‘anti‐identity’. This implies that ‘old’ dichotomies between physicians and managers may be gradually supplemented or replaced with ‘new’ dichotomies between ‘leading’ and ‘detached’ physicians.

We argue that our specific approach of observing a MLDP reveals valuable insights into *collective* identity work, thereby contributing to recent studies in the professional identity literature (Kyratsis *et al*. 2017, McGivern *et al*. 2015, Pouthier *et al*. 2013, Reay *et al*. 2017). However, one could question to what extent the move towards a collaborative identity was solely caused by the MLDP programme given the limited time span of the programme (1 year). We argue that the programme cannot be seen as entirely decoupled from practice. Rather, we argue that this move is a result from interactions between practice and the programme and is caused by a variety of factors. Collective discussions during plenary sessions, the in‐house sessions, practical experiences concerning individual improvement projects, feedback from facilitators or hospital boards and discussions with peers in daily medical practice all together fuelled a move towards a more collaborative understanding of leadership. Further research could therefore consider longer periods of observations *in situ*, including shadowing physicians in their daily work, to gain a better understanding of identity processes of physicians in hospitals.

## Appendix 1 Content of the program and examples of individual improvement projects

**Table:**

Content of the program	Examples of individual improvement projects
**Module 1:** Introduction, theory on leadership styles, group discussion about medical leadership, personal reflection exercise	Establishing a multidisciplinary breast centre
**Module 2:** Theory on quality dimensions, theory on short‐cycle improvements, individual exercise in applying these on personal projects	Educating new professionals, for example, a specialised nurse at the maternity ward
**Module 3:** Guest speaker on leadership in ‘high performance organisations’, exercise in applying insights on personal projects	Harmonising care protocols among different hospital locations
**Module 4:** Guest speaker (physician) on personal leadership, guest speaker (psychologist) on relations in teams, session with directors on lifestyle and prevention	Improving emergency care by increasing collaboration with primary care
**Module 5:** Guest speakers (director top‐clinical hospitals, patient) on shared decision‐making, guest speaker (director Dutch inspectorate) on policy‐making and accountability, guest speaker (financial advisor) on healthcare costs and efficiency**,** expert panel healthcare entrepreneurs	Developing a medical app (i.e. an app for pregnant women in which they can find personal information from midwives, gynaecologists and about maternity care)
**Module 6:** Guest speakers (two physicians, one nurse) about their role as ‘medical leader’, guest speaker (director Dutch Council for Public Health and Society)	Coordinating hospital‐broad value‐based healthcare projects
**In‐house sessions** (3): participants present the developments of their individual improvement projects to hospital directors, facilitators and peers	Establishing a disease‐specific network among different disciplines, professions and organisations

## Appendix 2 Example of the narratives *[quotes are from participants, unless indicated otherwise]*

**Table:**

**Heroic Narrative** *‘the pioneer’*		
My goal as a leader is to convince others of my vision As a leader I think it is extremely important to get along the laggards I want to be an inspiration to others	Visionary	Shaping the context
Someone who is leading the fight. People who take up actions A medical leader is someone who can master burn‐outs and who doesn't drown in the amount of work	Hard worker	
Not everyone is capable of being a medical leader It's time for a new (and young) generation	Happy few	
A medical leader is not only outstanding in her/his own medical specialty, but also takes responsibility in continuously improving patient care (program's brochure)	More than the clinical	
Someone who has organised patient care perfectly You are a good doctor and therefore you are going to lead	Medical excellence	
**Clinical Narrative ** *‘the patients’ guardian’*		
First comes the work floor, then finance and then profits. That's what makes us special You are the autonomous professional. You know that. You will make the difference, not the managers, not the board of directors (guest speaker)	Specialness	Disconnecting from the context
There are more and more suits in the cafeteria. I don't like it, we should keep all those guru's outside the hospital The board of directors has a hidden agenda. They just want everything cheaper and more efficient They [managers] don't have any insights about quality of care. They only care about costs	Not‐manager	
What distinguishes a medical leader is the connection to the patient You [physician] don't work primarily for the organisation. That is not your primary concern No one says I want to be a medical leader because I want to increase production	Disconnected	
We [rather than managers] really feel what people go through in the consultation room A medical leader is someone who is part of the physicians	Exclusive	
Our role is to improve patient care, that's different than [medical] managers, who are occupied with work schedules, performance, finance etc. When I pick up a patient from the waiting room I really don't care about any costs	Quality of care	
**Collaborative Narrative ** *‘the linking‐pin’*		
You have to do it together that is a characteristic of a medical leader. Learning to speak each other's language. Learning to transcend your own discipline	Inclusive	Adjusting the context
I used to consider expressing emotions on the work floor as unprofessional behaviour, now I realize I was wrong Peer support and open communication are important subjects what we as a young generation should stimulate to express more	Reflexive	
Everyone is shouting ‘patient first’ yet no one acts like it. We must leave behind our own blood group [medical specialty]	Multi‐disciplinary	
If you want to be an entrepreneur, you should have financial knowledge A medical leader should be responsible for both quality and costs (guest speaker)	Cost‐efficiency	
Hospital directors, medical staff directors and medical specialists all have to face the same challenge: how can we improve health care through efficient use of resources? (program's brochure)	Emphasizing interdependencies	

## Appendix 3 Examples of identity *un*doing

**Table:**

**Identity undoing**	
***Deconstruction of the heroic leader identity***	
**Tension: unable to engage others in adopting a vision**	**Deconstruction**
‘I developed an application for pregnant women in which they can follow their own care trajectory. […] So now my goal is to convince the midwives. [Then thinks deeply.] Yes, as a leader I think it is extremely important to get along the laggards. You see, I know what I want, but is much more important that I convince others too. Midwives can be very conservative’. (Respondent 3, in‐house session 15 January 2018)	‘What really helps me in projects where everyone holds a different view is to ask them ‘what do you need to get attached? And how can I help you?’ You really have to realize that it is a new game you are playing. A very difficult game’. (Hospital director)
‘There are people in our organization … they just do not care at all. I found that extremely difficult …’ (Respondent 19, module 3)	‘Choose your battles. There are people who do listen [name respondent 19]. Who do want to change’. (Guest speaker 4)
‘Trust is lacking [in my project]. That's the big issue. I'm standing still for two years now’. (Respondent 3, module 4)	‘Ok, but what is your circle of influence? You all have the ambition to put the patient central. […] So which people do you need to address? And how?’ (Guest speaker 5)
‘Do you have a tool for pure reluctance? But when you cannot fire them’. [everyone laughs loudly] (Respondent 18, module 3)	‘The answer is attention. […] So, [name respondent 18] trust me, it's also because of you that those others do not want to change. So have a look at yourself too’. (Guest speaker 4)
‘Sometimes I just wish that someone just take a decision instead of having to argue over and over again and have another three meetings about the same issue’ (Respondent 11, module 2)	‘But you can also turn it around right. We wanted to change the mamma clinic into a multidisciplinary center. Every physician was reluctant. But then we asked PR to interview everyone, the radiologists, the pathologists. Slowly everyone became enthusiastic, you have to create a feeling of ownership. It can help to just give other people credits’. (Respondent 6)
***Deconstruction of the clinical leader identity***	
**Tension: others influence professional work**	**Deconstruction**
‘I noticed that [name Michelin‐star restaurant] created their own quality norms. Compared to hospitals, so many quality norms are imposed upon us by outsiders …’ (Respondent 3, module 3)	‘Mister Michelin is just one norm, but we don't think this is high enough. So, how do you deal with norms that are imposed upon you from the outside? I think that you really need to know *yourselve*s that you deliver high quality of care and determine your own norms’. (Guest speaker 4)
‘External parties determine how you work and you have almost no influence on that’. (Respondent 18, module 5)	‘And therefore it is so important to acquire knowledge of your external environment. There is so much knowledge available, but maybe you don't possess it yet’. (Guest speaker 2)‘I thought that the hospital board would deal with that, but I guess I shouldn't count on that’. (Respondent 9)
‘Our department is a flat organization, like a family. Who doesn't like that is the hospital. It is extremely hard for them to involve with us. So what you get is that managers are trying to do stuff behind our backs. And that causes friction’. (Respondent 19, module 3)	‘You reign too much in your bastions and take too little notice of your surroundings. I would hate you too. And I blame you for the consequences. […] Although you think you are accessible… you are not, and so there is friction’. (Guest speaker 4)
‘It is often a battle between managers and physicians. Managers don't have any insight into quality of care. Only into costs. So a medical leader has to do both. Causes a lot of tensions. Collaboration is very difficult. […] They are not the ideal partner’. (Respondent 20, module 5)	‘I don't really recognize this. If you have an experienced business manager. […] We are lucky with business managers with backgrounds in nursing and they have insights in both quality and costs. […] There is often more room than you think there is’. (Respondent 24)
‘I am extremely bothered by managers. We're just not like minded. […] They have their budget and I see my patients and those are two totally different worlds. I don't know what he does and he doesn't have a clue what I am doing’. (Respondent 18, module 4).	‘I get the creeps from managers too. But I do sit down with business managers very consciously because they often are the key to logistics [in change projects]’. (Respondent 6)
